# eccDB: a comprehensive repository for eccDNA-mediated chromatin contacts in multi-species

**DOI:** 10.1093/bioinformatics/btad173

**Published:** 2023-04-05

**Authors:** Min Yang, Bo Qiu, Guo-You He, Jian-Yuan Zhou, Hao-Jie Yu, Yu-Ying Zhang, Yan-Shang Li, Tai-Song Li, Jin-Cheng Guo, Xue-Cang Li, Jian-Jun Xie

**Affiliations:** Guangdong Provincial Key Laboratory of Medical Molecular Diagnostics, Institute of Aging Research, Guangdong Medical University, Dongguan 523808, China; Institute of Biochemistry & Molecular Biology, Guangdong Medical University, Zhanjiang 524023, China; Department of Pathology, Medical College of Jiaying University, Meizhou 514031, China; Department of Medical Informatics, Harbin Medical University, Daqing Campus, Daqing 163319, China; Department of Medical Oncology, Qilu Hospital, Cheeloo College of Medicine, Shandong University, Jinan 250012, China; Department of Medical Informatics, Harbin Medical University, Daqing Campus, Daqing 163319, China; Department of Biochemistry and Molecular Biology, Medical College of Shantou University, Shantou 515063, China; Department of Biochemistry and Molecular Biology, Medical College of Shantou University, Shantou 515063, China; Department of Biochemistry and Molecular Biology, Medical College of Shantou University, Shantou 515063, China; School of Traditional Chinese Medicine, Beijing University of Chinese Medicine, Beijing 100029, China; Department of Medical Informatics, Harbin Medical University, Daqing Campus, Daqing 163319, China; Guangdong Provincial Key Laboratory of Medical Molecular Diagnostics, Institute of Aging Research, Guangdong Medical University, Dongguan 523808, China; Institute of Biochemistry & Molecular Biology, Guangdong Medical University, Zhanjiang 524023, China

## Abstract

**Summary:**

We developed the eccDB database to integrate available resources for extrachromosomal circular DNA (eccDNA) data. eccDB is a comprehensive repository for storing, browsing, searching, and analyzing eccDNAs from multispecies. The database provides regulatory and epigenetic information on eccDNAs, with a focus on analyzing intrachromosomal and interchromosomal interactions to predict their transcriptional regulatory functions. Moreover, eccDB identifies eccDNAs from unknown DNA sequences and analyzes the functional and evolutionary relationships of eccDNAs among different species. Overall, eccDB offers web-based analytical tools and a comprehensive resource for biologists and clinicians to decipher the molecular regulatory mechanisms of eccDNAs.

**Availability and implementation:**

eccDB is freely available at http://www.xiejjlab.bio/eccDB.

## 1 Introduction

Circular DNA molecules, or extrachromosomal circular DNAs (eccDNAs), are detached from chromosomal presence (Hotta and Bassel 1965; Zuo et al. 2022) and carry genes that are highly expressed in cancer with poor prognosis. Furthermore, eccDNAs interact with chromosomes and have been suggested to play a key role in transcriptional regulation (Paulsen et al. 2018; Wu et al. 2019; Kim et al. 2020; Zhu et al. 2021).

Due to the role of eccDNAs in gene expression and transcriptional regulation, we have developed the eccDB, which is a comprehensive repository for eccDNA-mediated chromatin contacts in multispecies. eccDB focuses on providing a large number of available resources for eccDNAs across multiple species, including *Homo sapiens*, *Mus musculus*, *Saccharomyces cerevisiae*, and *Arabidopsis thaliana*. eccDB provides multiple analysis of eccDNAs, such as gene expression, functional enrichment, regulatory elements annotation. In addition, eccDB can help users to analyze eccDNAs intrachromosomal and interchromosomal interactions, or analyze whether the unknown sequence is similar to eccDNAs. Overall, eccDB is a comprehensive repository for storing, browsing, searching, and analyzing eccDNAs.

## 2 Materials and methods

### 2.1 eccDNA collection and identification

**Figure 1:**
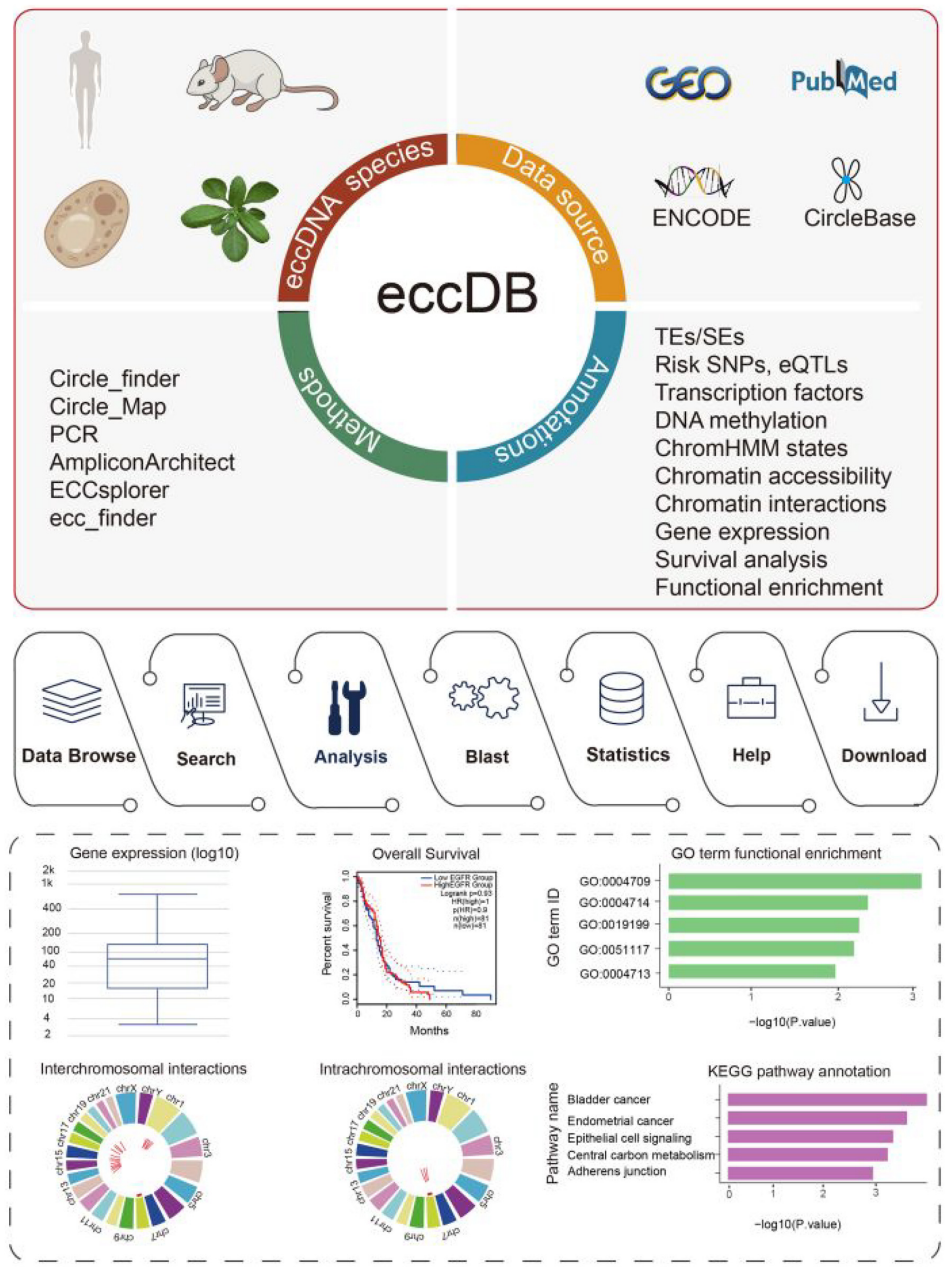
eccDB content and construction. eccDB uses a combination of identification methods to identify eccDNAs in four species (*Homo sapiens*, *Mus musculus*, *Saccharomyces cerevisiae*, and *Arabidopsis thaliana*) and collects multiomics data from different resources. eccDB annotates eccDNAs regulatory elements, and genetic and epigenetic information including TEs/SEs, Risk SNPs, eQTLs, TFs, DNA methylation, ChromHMM states, and Chromatian accessibility. eccDB offers a set of tools to explore eccDNAs, including data browse, search, blast, and analysis. In analysis, eccDB provides gene expression analysis, survival analysis, GO term functional enrichment, KEGG pathway annotation, and intrachromosomal and interchromosomal interaction analysis.

The eccDB contains eccDNAs obtained by multiple methods, mainly including Circle_finder (Dillon et al. 2015; Kumar et al. 2017), Circle_Map (Prada-Luengo et al. 2019), and literature mining. Part of human eccDNAs was downloaded from CircleBase (Zhao et al. 2022). Finally, the eccDNAs with length <50 MB are retained (Fig. 1). The number of samples and eccDNAs for each species are shown in Supplementary Table S1.

### 2.2 eccDNA annotations

To annotate the regulatory elements of eccDNAs, we downloaded a large number of regulatory elements from public databases, including typical-enhancers (TEs), super-enhancers (SEs), transcription factors (TFs), DNA methylation, risk SNPs, eQTLs, chromatin accessibility, and chromHMM states. The list of public databases is in Supplementary Table S2. Besides, the eccDB further categorized the eccDNA genes into three different types and divided the eccDNAs into four distinct categories (Møller et al. 2020; Ling et al. 2021).

### 2.3 eccDNA chromosomal interaction

We proposed a method for predicting eccDNA and genome-wide chromosomal interactions, which is based on sequence similarity and chromosomal interaction data. To accomplish this, our study implemented ChIA-PET, 3C, and Hi-C data (Teng et al. 2015), along with BLAST+ (ncbi-blast-2.13.0-x64-linux) to conduct sequence similarity analysis.

The eccDNAs chromosomal interactions that were identified through this method were classified into two categories: intrachromosomal and interchromosomal interactions. Intrachromosomal interactions indicate when an eccDNA interacts with a fragment from the same chromosome, while interchromosomal interactions refer to when an eccDNA interacts with a fragment from a different chromosome.

More detailed description of the method and parameters are on the “Help” page.

## 3 Results

### 3.1 eccDB overview and current content

The eccDB offers a wide range of query methods, including “Search eccDNA by gene name”, “Search eccDNA by human disease type”, “Search eccDNA by method”, “Search eccDNA by genome region”, “Search eccDNA by tissue type”, and “Search eccDNA by eccDNA ID”.  Additionally, it supports eccDNAs gene expression analysis, survival analysis, GO term functional enrichment analysis, and KEGG pathway annotation, allowing users to gain a better understanding of eccDNAs.

The eccDB also provides regulatory and epigenetic information annotations for eccDNAs, which are presented in both tabular and graphical. Moreover, it offers sequence similarity analysis of eccDNA in four species: *H.sapiens*, *M.musculus*, *S.cerevisiae*, and *A.thaliana*.

What makes the eccDB unique is its ability to provide predictions for both intrachromosomal and interchromosomal chromosomal interactions of eccDNAs, utilizing DNA sequence similarity and chromosome interaction data. Additionally, eccDNAs information on the database is available for free download (Fig. 1).

### 3.2 eccDB usage

To demonstrate how to use eccDB, we provide an example which includes browsing samples information, searching for eccDNAs, viewing regulatory elements information about eccDNAs, and more (Supplementary Fig. S1). Furthermore, two effective examples are given to illustrate how to use eccDB to identify potential chromosome interactions and predict the function of eccDNAs (Supplementary File, Supplementary Figs S2 and S3).

## 4 Conclusions

eccDB offers a user-friendly interface for users to query, analyze, browse, and visualize detailed information about eccDNAs. The key advantages of eccDB include: (i) a comprehensive repository for eccDNAs; (ii) sequence similarity analysis of eccDNAs; and (iii) interchromosomal and intrachromosomal interaction analysis. All of this allows researchers to gain insight into the mechanisms of occurrence and potential biological functions of eccDNAs.

## Supplementary Material

btad173_Supplementary_Data

## Data Availability

eccDNA information is available for free download at eccDB (http://www.xiejjlab.bio/eccDB).
