# Supplementary material for: eccDB: a comprehensive repository for eccDNA-mediated chromatin contacts in multi-species
Source: Bioinformatics. 2023 Apr 5;39(4):btad173. doi: 10.1093/bioinformatics/btad173 (PMC10112955; doi:10.1093/bioinformatics/btad173)
Supplement: btad173_Supplementary_Data [file btad173_supplementary_data.zip › Supplementary Figure 2.pdf]

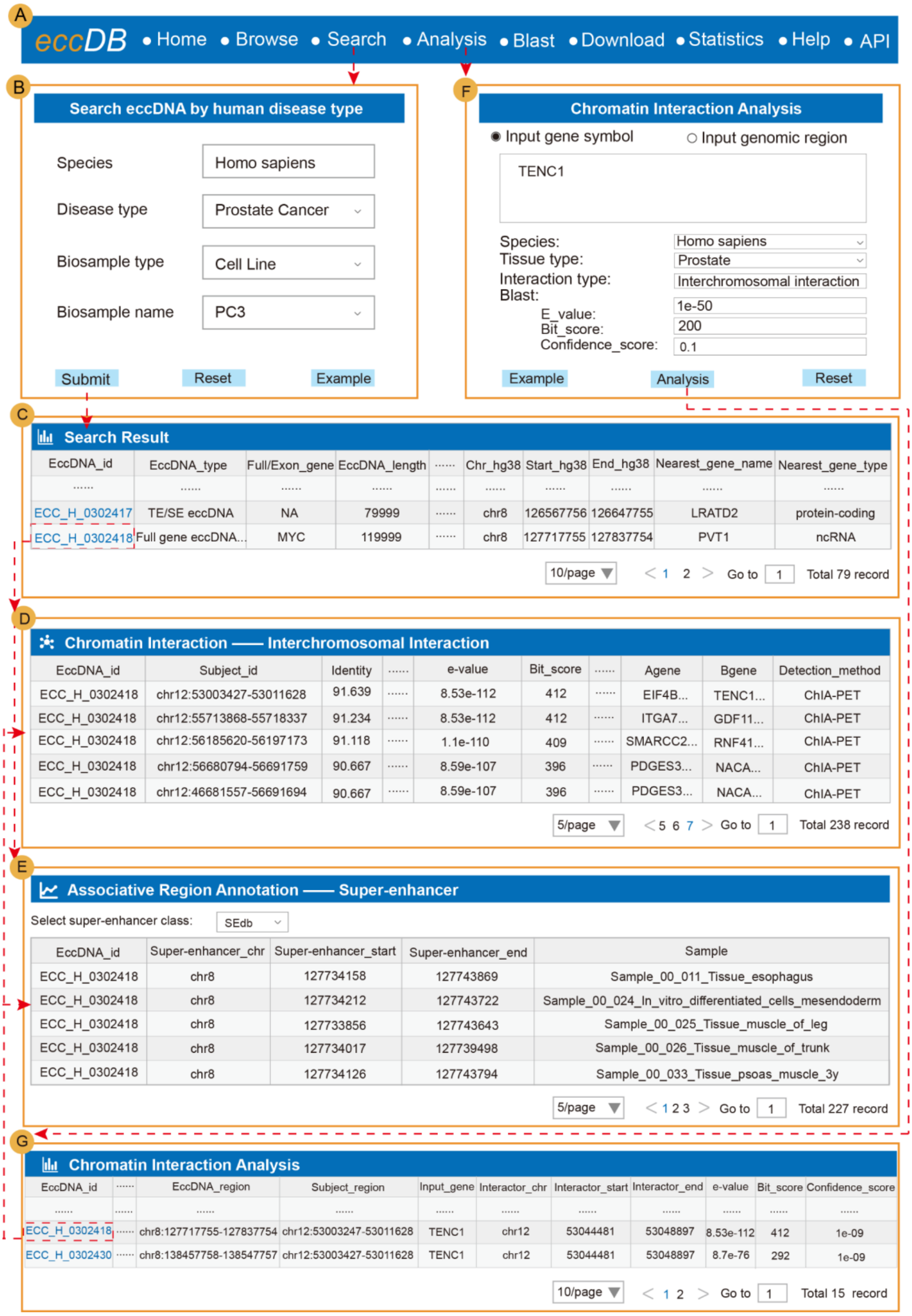

Supplementary Figure 2. Case study for eccDNA chromosomal interaction and analysis. (A) Top navigation bar help users use functions of this database. (B) Search eccDNA by human disease type (Disease type: Prostate Cancer; Biosample type: Cell Line; Biosample name: PC3) as an example. (C) List of eccDNA searched by human

disease type (set search criteria by Supplementary Figure 2B). (D) List of “ECC_H_0302418” interchromosomal interaction. (E) List of “ECC_H_0302418” super-enhancer from SEdb. (F) The chromosome interaction analysis of *TENC1* gene, set species as Homo sapiens, tissue type as Prostate, interaction type as interchromosomal interaction, and Blast as default value. (G) The result of *TENC1* interchromosomal interaction analysis.
