# Supplementary material for: eccDB: a comprehensive repository for eccDNA-mediated chromatin contacts in multi-species
Source: Bioinformatics. 2023 Apr 5;39(4):btad173. doi: 10.1093/bioinformatics/btad173 (PMC10112955; doi:10.1093/bioinformatics/btad173)
Supplement: btad173_Supplementary_Data [file btad173_supplementary_data.zip › Supplementary Figure 3.pdf]

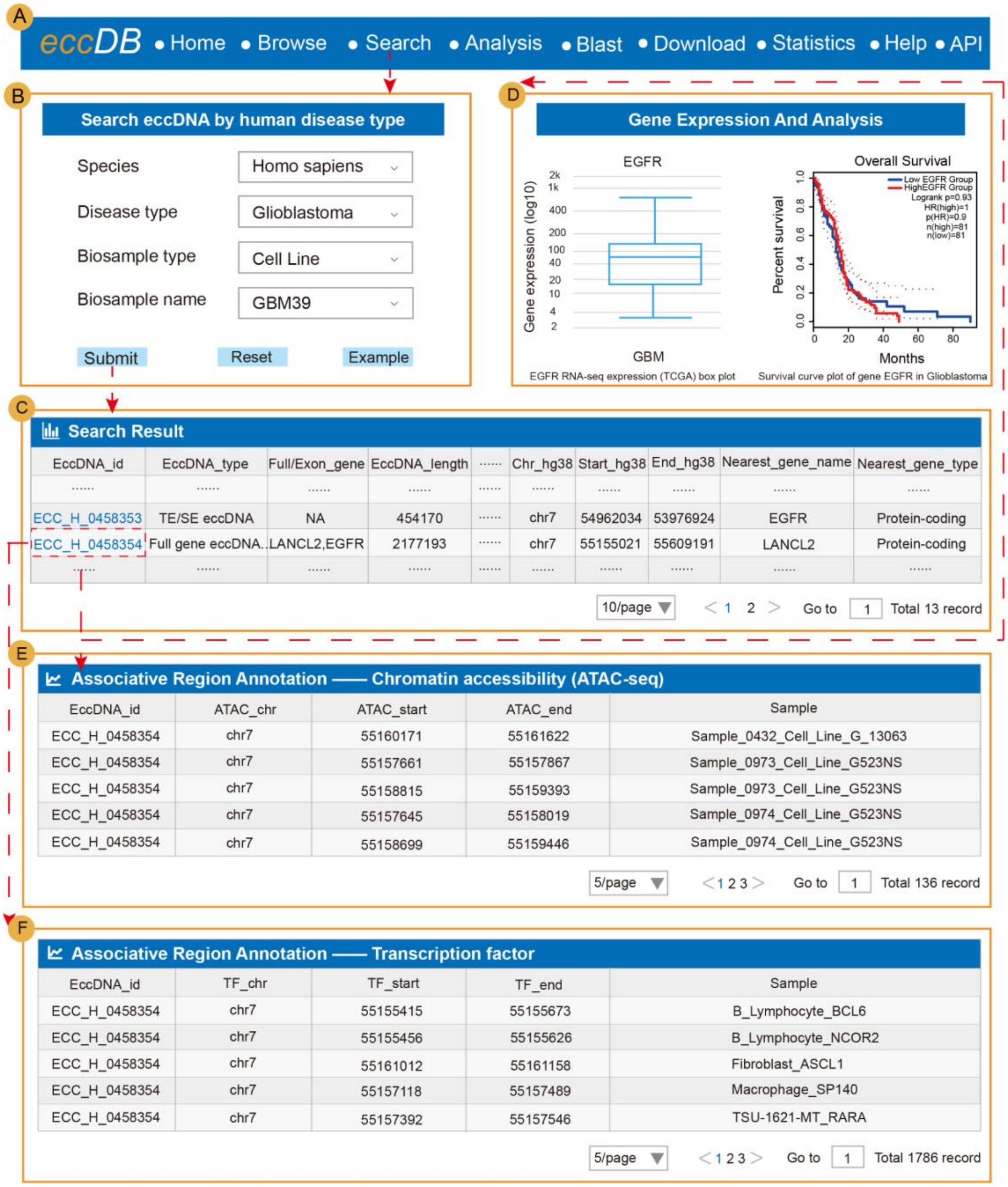

Supplementary Figure 3. Case study for eccDNA transcription. (A) Top navigation bar help users use functions of this database. (B) Search eccDNA by human disease type (Disease type: Glioblastoma; Biosample type: Cell Line; Biosample name: GBM39). (C) List of eccDNA searched by human disease type (set search criteria by Supplementary Figure 3B). (D) On the left, the expression of *EGFR* (TCGA) in Glioblastoma (GBM) tumor samples with box plots, and the survival curve of *EGFR* in GBM on the right. (E) Information about the chromatin accessibility regions of "ECC_H_0458354". (F) Information about the transcription factors of

“ECC_H_0458354”.
