# Supplementary material for: eccDB: a comprehensive repository for eccDNA-mediated chromatin contacts in multi-species
Source: Bioinformatics. 2023 Apr 5;39(4):btad173. doi: 10.1093/bioinformatics/btad173 (PMC10112955; doi:10.1093/bioinformatics/btad173)
Supplement: btad173_Supplementary_Data [file btad173_supplementary_data.zip › Supplementary File.pdf]

Case study for eccDNA chromosomal interaction analysis

Multi-genomic analysis of eccDNA-associated chromosomal genes, SE features, and RNA expression demonstrated that eccDNAs can act as mobile transcriptional SE elements to promote tumor progression and showed a potential transcriptional regulatory mechanism for synthetic aneuploidy. Zhu Y *et al.* adopted ChIA-Drop assays and identified eccDNA-chromosome interactions in the PC3 human prostate cancer cell line (Zhu et al. 2021). To exemplify the accuracy of eccDB for collecting information on eccDNA-chromosome interactions, we used the "Search eccDNA by human disease type" (Disease type: Prostate cancer; Biosample type: Cell Line; Biosample name: PC3) as search criteria (Supplementary Figure 2B). The resulting output table displayed concise and informative annotations for each eccDNA identified in the PC3 cell line. (Supplementary Figure 2C). Taking "ECC_H_0302418" as an example, we can see that the eccDNA "ECC_H_0302418" carries the *MYC* gene. SEs are a large cluster of transcriptionally active enhancers that play a key role in the identity of human cells in health and disease, driving gene expression (Hnisz et al. 2013). eccDB shows that "ECC_H_0302418" has 227 SE regions from SEdb database (Supplementary Figure 2E), which has potentially high transcriptional activity. Similarity analysis of DNA sequence is a necessary process to compare unknown DNA sequences with known ones for inferring the functions of unknown ones (Wang et al. 2010). Therefore, eccDB provides chromosomal interaction prediction based on DNA sequence similarity and chromosome interaction data (including ChIA-PET, 3C, and Hi-C). And the eccDNA "ECC_H_0302418" has 238 interchromosomal interactions and 7 intrachromosomal interactions (Supplementary Figure 2D).

Additionally, eccDB identified sequence similarity between "ECC_H_0302418" and the *EIF4B* gene (chr12:53003427-53011628, e_value = 8.53e-112, bit_score = 412). In "Chromatin Interaction", the table of interchromosomal interaction shows that the *TENC1* and *EIF4B* interaction was verified by ChIA-PET. Therefore, we inferred that "ECC_H_0302418" and *TENC1* have a potential interaction relationship based on sequence similarity (Supplementary Figure 2D). The "ECC_H_0302418" also interacts with other genes, such as *RNF41*, *CSDE1*, *ATP5B*, and *CIRBP*. These genes can be queried in the list of genes interacting with eccDNA detected by Zhu Y *et al*. (Zhu et al. 2021).

Furthermore, when we sequentially entered genes *TENC1*, *RNF41*, *CSDE1*, *ATP5B*, and *CIRBP* on the "Analysis" page, set species as Homo sapiens, tissue type as Prostate,

interaction type as interchromosomal interaction, and blast section as default parameters for analysis (Supplementary Figure 2F). The result showed that all these genes interacted with “ECC_H_0302418”. This indicates that the results of the “Analysis” are mutually validated. And the “Analysis” can be used to predict the interaction between eccDNAs and chromosomes (Supplementary Figure 2G).

Case study for eccDNA transcription

In cancer, oncogenes are commonly amplified on eccDNAs. Recent studies have shown that oncogenes encoded by eccDNAs are highly expressed in the transcriptome of tumors, indicating their significant contribution to the development of cancer (Paulsen et al. 2018; Kim et al. 2020). Moreover, research has demonstrated that eccDNAs exhibit drastically increased chromatin accessibility, suggesting their potential involvement in tumor progression (Wu et al. 2019).

To search for eccDNAs associated with Glioblastoma (GBM), we used the “Search eccDNA by human disease type” tool. By inputting Disease type: Glioblastoma, Biosample type: Cell Line, and Biosample name: GBM39 (Supplementary Figure 3B), we were able to identify eccDNA “ECC_H_0458354” (Supplementary Figure 3C). This eccDNA carried the *LANCL2*, *VOPP1*, and *EGFR* genes, with EGFR being an oncogene. The expression of *EGFR* in Glioblastoma tumor samples was shown using box plots from eccDB based on TCGA data (Supplementary Figure 3D, left). Moreover, eccDB provided survival analysis that demonstrated a lower survival rate for Glioblastoma patients with high *EGFR* expression (Supplementary Figure 3D, right). Based on these findings, we can infer that patients carrying “ECC_H_0458354” are likely to have a poorer prognosis due to the presence of the oncogene *EGFR*.

Studies have shown that *EGFR* gene is highly expressed in GBM and amplification of the *EGFR* gene was associated with a poorer prognosis (Xu et al. 2018; Layfiled et al. 2016). Interestingly, Wu S *et al.* found that oncogenes encoded on eccDNA (including *EGFR*, *MYC*, *CDK4*, and *MDM2*) were highly expressed in the cancer genome (Wu et al. 2019). eccDNAs carried *EGFR* gene was identified in patient-derived GBM cell lines by Kumar P *et al.* The *EGFR* gene is amplified through the formation of eccDNA in GBM (Kumar et al. 2020). Several studies have shown that eccDNAs play a role in the upregulation of EGFR, a finding that is supported by our own database. Furthermore, we have identified 136 regions of chromatin accessibility and 1,786 TF binding sites on the "ECC_H_0458354" eccDNA (Supplementary Figure

3E, F), providing further evidence of the significant enhancement of chromatin accessibility observed in this specific eccDNA in cancer.

References

Hnisz, D. *et al*. (2013) Super-enhancers in the control of cell identity and disease. *Cell*. 2013;155(4):934-947.

Kim, H. *et al*. (2020) Extrachromosomal DNA is associated with oncogene amplification and poor outcome across multiple cancers. *Nat Genet*. 2020;52(9):891-897.

Kumar, P. *et al*. (2020) ATAC-seq identifies thousands of extrachromosomal circular DNA in cancer and cell lines. *Sci Adv*. 2020;6(20):eaba2489.

Layfield, LJ. *et al*. (2006) Epidermal growth factor receptor gene amplification and protein expression in glioblastoma multiforme: prognostic significance and relationship to other prognostic factors. *Appl Immunohistochem Mol Morphol*. 2006;14(1):91-96.

Paulsen, T. *et al*. (2018) Discoveries of Extrachromosomal Circles of DNA in Normal and Tumor Cells. *Trends Genet*. 2018;34(4):270-278.

Wang, S. *et al*. (2010) Bilateral similarity function: a novel and universal method for similarity analysis of biological sequences. *J Theor Biol*. 2010;265(2):194-201.

Wu, S. *et al*. (2019) Circular ecDNA promotes accessible chromatin and high oncogene expression. *Nature*. 2019;575(7784):699-703.

Xu, G. *et al*. (2018) CDK4, CDK6, cyclin D1, p16(INK4a) and EGFR expression in glioblastoma with a primitive neuronal component. *J Neurooncol*. 2018;136(3):445-452.

Zhu, Y. *et al*. (2021) Oncogenic extrachromosomal DNA functions as mobile enhancers to globally amplify chromosomal transcription. *Cancer Cell*. 2021;39(5):694-707.e7.
