# Supplementary material for: eccDB: a comprehensive repository for eccDNA-mediated chromatin contacts in multi-species
Source: Bioinformatics. 2023 Apr 5;39(4):btad173. doi: 10.1093/bioinformatics/btad173 (PMC10112955; doi:10.1093/bioinformatics/btad173)
Supplement: btad173_Supplementary_Data [file btad173_supplementary_data.zip › Supplementary Figure 1.pdf]

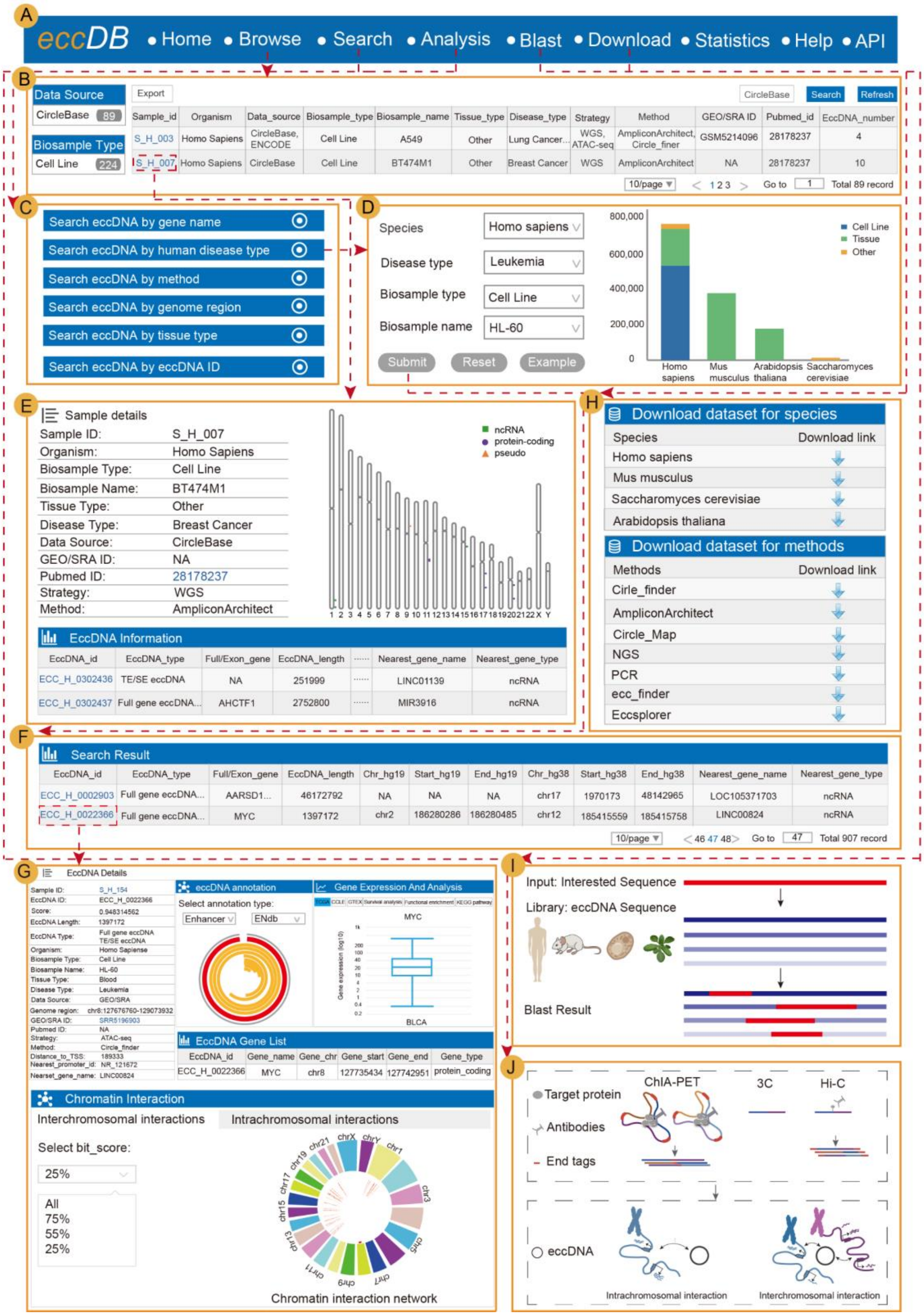

Supplementary Figure 1. The main functions and usage of eccDB. (A) The navigation bar of eccDB. (B) The eccDB "Browse" page to view sample brief information in four species. (C) The eccDB provides six query methods for searching eccDNA: "Search eccDNA by gene name", "Search eccDNA by human disease type", "Search eccDNA

by method”, “Search eccDNA by genome region”, “Search eccDNA by tissue type” and “Search eccDNA by eccDNA ID”. (D) Taking “Search eccDNA by human disease type” as an example, users select the disease type, biosample type, and biosample name of the human of interest. (E) The presented interface delivers a comprehensive overview of the sample detail information. (F) List of eccDNA searched by human disease type (Disease type: Leukemia; Biosample type: Cell Line; Biosample name: HL-60). (G) This interface is the eccDNA details page and describes the eccDNA sample information, eccDNA type, eccDNA gene list, eccDNA gene expression analysis and analysis (including survival analysis, GO term functional enrichment analysis, and KEGG pathway annotation), eccDNA chromatin interaction, and eccDNA annotation information (TEs, SEs, Chromatin accessibility regions, ChromHMM states, TFs, DNA methylation positions, Risk SNPs, eQTLs). (H) The “Download” allows users to download eccDNAs information by species, or eccDNAs information by methods. (I) The eccDB helps users analyze whether the nucleic acid sequence of interest has sequence similarity to eccDNA in four species. (J) The eccDB provides eccDNA intrachromosomal and interchromosomal interactions analysis.
